# DNA Barcoding Protocol for *Masdevallia* Orchids: A Tool for CITES-Based Identification and Trade Control

**DOI:** 10.3390/genes16121423

**Published:** 2025-11-29

**Authors:** Małgorzata Karbarz, Eliza Lebioda, Agnieszka Leśko

**Affiliations:** 1 Faculty of Biology, Nature Protection, and Sustainable Development, University of Rzeszów, 35-601 Rzeszów, Poland; 2 Muzeum-Zamek w Łańcucie, 37-100 Łańcut, Poland

**Keywords:** *Masdevallia*, DNA barcoding, identification, orchids

## Abstract

**Background**: Orchids of the *Masdevallia* genus are characterized by their beautiful esthetic qualities. However, they are vulnerable to habitat destruction, illegal harvesting, tourism and climate change. These extinction threats have led to the listing of all *Masdevallia* species in Appendix II of CITES (Convention on International Trade in Endangered Species of Wild Fauna and Flora) and their selection by the IUCN (International Union for Conservation of Nature). Orchids are sold in various forms, e.g., stems or tubers, rendering them impossible to identify based on morphology alone. DNA barcoding is a method that enables reliable identification of organisms using DNA barcodes, and it offers an excellent solution to the need for efficient identification of *Masdevallia* orchids. The aim of this study was to determine the most effective locus for DNA barcode identification of *Masdevallia* orchids in order to develop a quick and practical identification method for this genus. Such a method can be used by CITES verification authorities to detect illegal trade. This is the first focused study to validate a DNA barcoding protocol for *Masdevallia* for CITES enforcement purposes. **Methods**: Three genetic regions were analyzed: *matK*, *rbcL*, and ITS. The effectiveness of identification was verified based on results obtained from the new version of the BOLD Systems v.5 reference database. **Results**: Although *Masdevallia* is not well represented in this database, successful identification to the genus level was achieved. **Conclusions**: The highest identification efficiency at the genus level was achieved for the ITS region (91%).

## 1. Introduction

DNA barcoding is a method that involves identifying a code consisting of one or more short DNA sequences in a selected region of the nuclear or organelle genome that is characteristic of a given species. This code is called a DNA barcode. Their analysis enables the identification of previously described as well as newly discovered species [1]. The term “DNA barcoding” was first introduced in 2003 by Paul Hebert [2]. The DNA barcoding procedure includes: material collection, DNA isolation from the collected material, barcode region amplification, electrophoresis, sequencing, and comparison of the obtained sequences with a database. Cox1 is considered the standard barcode for animals and ITS for fungi [3]. Many researchers have concluded that multiple markers are necessary to achieve adequate species discrimination in plants [4]. Recent advances in chloroplast phylogenomics highlight the advantages of using complete chloroplast (cp) genomes over single-marker approaches for DNA barcoding, offering higher resolution for species identification and robust phylogenetic inference [5,6]. In Dalbergia, highly variable regions within cp genomes provide valuable plastid markers for identification, phylogeny, and population genetics, including applications in monitoring species intercepted in illegal trade [6].

DNA barcoding, despite its wide use, has important limitations. Its accuracy depends on complete reference databases, and gaps can lead to misidentifications [7]. Hybridization, ancestral polymorphisms, and poor sample quality further hinder precise species identification [7,8,9].

*Masdevallia* (Ruiz & Pav.) belongs to the family Orchidaceae (orchids). The genus comprises 652 accepted species, including 9 hybrids. *Masdevallia* originate from South and Central America [10]. They are characterized by often brightly colored flowers and long, petiolate sepals with small petals. The leaves are smooth and fleshy [11]. This genus was first reported by Ruiz & Pavón in 1794 [10]. About 100 species are considered endemic to Peru, and there are even taxa that are known from only one region or locality [12]. Currently (data from 2025), only 11 species of *Masdevallia* are listed on the IUCN Red List of Threatened Species [13]. However, all *Masdevallia* species included in the CITES list are listed in Appendix II, which means that their trade must be controlled in order to avoid utilization incompatible with their survival. *Masdevallia* are not strongly represented in the Barcode of Life database, with a total of 94 recorded species, including 35 with barcodes—about 5% of all *Masdevallia* species [14,15,16].

*Masdevallia* have various applications, e.g., *M. picea*, *M. coccinea*, *M. stenorhynchos*, *M. herradurae*, *M. nidifica*, *M. civilis*, and *M. cuprea* are used in traditional medicine to treat fever, skin diseases and inflammation due to their antiseptic and anti-inflammatory properties, while *M. floribunda*, *M. coccinea*, *M. stenorchynchos*, *M. nidifica*, *M. cuprea*, and *M. schlimii* are popular in floristry and horticulture as ornamental plants [17]. Medicinal orchids are most often sold in the form of dried fragments, which makes their morphological identification difficult [18]. DNA barcoding enables the correct identification of such materials and supports the relevant institutions in controlling and monitoring illegal trade [18,19]. DNA barcoding is used to monitor both legal and illegal trade in wild species. It is particularly useful for identifying semi-processed or morphologically indistinguishable products [20]. Accurate identification at the species level helps to control overexploitation and enables the location of natural habitats to be determined and monitored for conservation purposes [18]. CITES has already begun the first practical applications of DNA barcoding, including in Pakistan, where DNA analysis identified a shipment falsely labeled as “fish meat” as containing Indian turtles (*Lissemys punctata*)—a species protected by the Convention [21]. The Barcode of Wildlife project is one of the first applications of DNA barcoding supported by CITES. Its aim is to create a public DNA sequence library for endangered species covered by the Convention, enabling more effective identification in cases of illegal trade. The project supports law enforcement and customs authorities in enforcing CITES regulations [22].

In Poland, the Department of Nature Conservation of the Ministry of the Environment is responsible for the implementation of the CITES Convention [23]. Enforcement involves controls at designated customs offices and border crossings, where shipments are checked for required certificates and permits [24]. In order to effectively combat illegal trade in wild animals and plants, forensic tools such as ballistics, DNA profiling and volatile organic compound profiling are used [25,26].

The current resolution emphasizes the crucial importance of accurate identification of plant and animal specimens in international trade in order to properly apply the provisions of the CITES Convention. It points out that identification may concern both species and higher taxonomic units (e.g., genus, family), as well as commercial products. The resolution draws attention to the diversity and development of identification methods, including electronic and molecular methods [27].

A special group called CBOL (Consortium for the Barcode Of Life), which develops plant barcodes, conducted research showing that the most reliable results for plants are obtained for chloroplast genes such as *matK* and *rbcL*. The *rbcL* gene, which has been sequenced in the largest number of plant species, was first used in phylogenetic studies. However, there were sometimes false results in the relationship between plants. Therefore, the *matK* gene, which provides more information than *rbcL*, is a good supplement [28]. The Consortium for the Barcode of Life (CBOL) has approved the *matK* and *rbcL* loci as official DNA barcodes for the identification of all terrestrial plants [29]. In 2025, the Barcode of Life consortium updated the BOLD database and added the ITS barcode as applicable for plant species identification [30].

The *matK* gene encoding maturase K is used as a marker for the identification of, among others, angiosperms, flowering plants and gymnosperms [31]. It more effective in species-level identification than *rbcL* [32]. However, it is more difficult to amplify using basic primers [33]. The *rbcL* gene cannot be used for species-level identification due to high homoplasy and low sequence variability [34,35]. The ITS gene has been used to identify over 21,000 plant species. The CBOL group does not recommend its use as a universal DNA barcode for plants, but as a complementary locus due to the possibility of different copies occurring. As a result of its greater discriminatory power and versatility, it is considered superior to chloroplast DNA barcodes [31]. Due to their diversity, the species identification of orchids is not straightforward, and usually requires the use of multi-barcodes [36,37].

Reference libraries are used for taxonomic identification. They contain DNA barcodes that have been assigned to previously identified taxa. There are several reference databases, including the largest reference library, BOLD Systems (Barcode of Life Data System), which has been developing rapidly for many years [38]. The latest available version, v.5, features a redesigned identification engine and an improved analytical compute cluster. The databases have also been expanded with additional DNA barcodes. Identification is now possible using: *COI-5P*, *rbcL*, *matK*, ITS, ITS1, ITS2, *18S*, *12S* and *CAD*. In addition, an operating mode function has been introduced, which allows for the selection of one out of three sets of settings, depending on the required speed, similarity threshold and desired number of hits. Depending on the selected mode, it is possible to send up to 1000 sequences at once, as compared to only 50 in v.4 [30]. The aim of the study was to determine which locus would be most effective as a DNA barcode for the identification of *Masdevallia* and to develop a rapid identification method that could be used by CITES enforcement authorities. To this end, we analyzed three genetic regions: *matK*, *rbcL*, and ITS. The effectiveness of identification was verified based on the results obtained in the latest reference database, BOLD Systems v.5. We aimed to identify the marker with the highest discriminatory potential for *Masdevallia*, which could support nature conservation efforts and serve as a basis for further research.

## 2. Materials and Methods

### 2.1. Sampling

**Figure 1:**
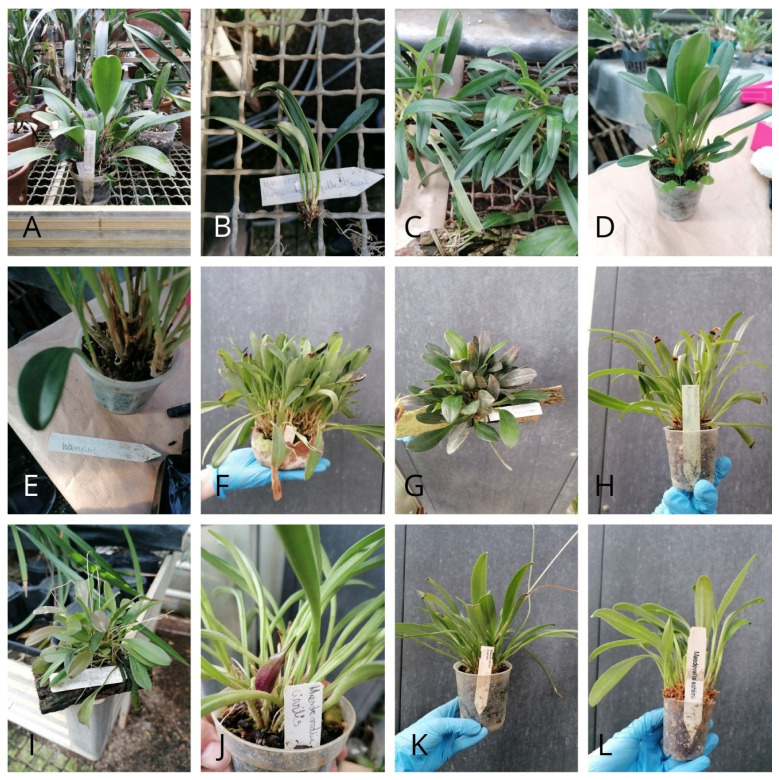
Examined species of *Masdevallia*. (**A**) *Masdevallia pelecaniceps* *, (**B**) *Masdevallia floribunda* var. *Galleottiana*, (**C**) *Masdevallia picea*, (**D**) *Masdevallia schroederiana*, (**E**) *Masdevallia coccinea* var. *harryana*, (**F**) *Masdevallia coccinea* f. *alba* Hermans, (**G**) *Masdevallia stenorhynchos*, (**H**) *Masdevallia herradurae*, (**I**) *Masdevallia nidifica*, (**J**) *Masdevallia civilis*, (**K**) *Masdevallia cuprea*, (**L**) *Masdevallia schlimii*. * Currently classified as *Phloeophila pelecaniceps*, it previously belonged to the genus *Masdevallia*.

The material was collected in the form of young leaves from all available *Masdevallia* species from the orchid collection belonging to the orchid house of Łańcut Museum-Castle (Figure 1). The fieldwork involved plant collection, data documentation and photography. Based on morphological characteristics, the specimens were preliminarily identified morphologically in collaboration with taxonomists.

### 2.2. DNA Isolation

The CTAB buffer method developed by Doyle [39] was used for total genomic DNA isolation from plants, following the procedure described by Karbarz et al. (2024) for *Paphiopedilum* orchids, with slight modifications [37]. In brief, 100 mg of young leaf tissue was homogenized in STE solution (0.25 M sucrose, 0.03 M Tris, 0.05 M EDTA). The homogenate was centrifuged and the pellet washed. DNA extraction was performed using a CTAB buffer, followed by purification with chloroform and precipitation with isopropanol. The DNA pellet was washed with 80% ethanol, dried, and dissolved in TE buffer. DNA concentration was measured using a NanoDrop ND-2000 spectrophotometer (Thermo Fisher Scientific Inc., Waltham, MA, USA).

### 2.3. Polymerase Chain Reaction (PCR) and Agarose Gel Electrophoresis

Primers: *matK* [40], *rbcL* [40] and ITS [40] were used to amplify the three barcode regions. PCR were carried out in 0.2 mL tubes with a reaction mixture that consisted of 1 µL of DNA, 1 µL of each primer (forward and reverse at a concentration of 10 μM), 10 μL of Taq PCR Master Mix (2×) (EURx Sp. z o.o., Gdańsk, Poland), and 7 μL of water, achieving final concentrations of 0.5 μM for each primer and 1× for the Master Mix in a total reaction volume of 20 μL. The reactions were conducted using a Labcycler Basic PCR thermocycler (Sensoquest, Göttingen, Germany). The thermocycler parameters for a given pair of primers are presented in Table 1. The table shows the initial denaturation, denaturation, annealing, elongation, and final elongation temperatures, as well as time and number of cycles. PCR products were separated on 1.5% agarose gels stained with Gelview and visualized using a Gel Doc XR + system (BioRad Richmond, VA, USA). A 50 bp DNA ladder was used as a size reference.

### 2.4. Sequencing and Data Analysis

Sanger sequencing was performed at the Laboratory of Molecular Biology Techniques at Adam Mickiewicz University in Poznań. The sequencing results were edited using BioEdit 7.2 software [41] to correct bases not identified by the program. Species and genus identification was based on a comparison of the sequences obtained with those deposited in the latest version of the BOLD Systems v.5 database. The analysis was performed by selecting the plant database and the rapid species search option, using the default settings (Search Depth: 94% similarity, Max Hits: 25 per sequence, Min Overlap: 100, Max Submission: 1000 sequences, average analysis time: 1 s/sequence) [42]. If only one species was identified and it matched the species of the analyzed orchid, the result was assigned to that species. If multiple species of the same genus were identified, the result was assigned to the genus. If multiple genera of a given subfamily were identified, the result was assigned to the subfamily.

## 3. Results and Discussion

This study is the first to develop a rapid and practical DNA barcoding protocol for *Masdevallia*, with a view to its application under the CITES Convention. Three genetic regions were analyzed—*matK*, *rbcL* and ITS—in order to determine their identification effectiveness.

PCR was used to amplify selected DNA regions: *matK*, *rbcL*, ITS for each species analyzed. The amplified products were then fractionated by gel electrophoresis. The highest amplification efficiency was obtained for the *rbcL* loci, amounting to 100%. The lowest efficiency was observed for the *matK* loci, with 83% efficiency for the analyzed orchid species. The amplification success rate for ITS was 92%. The lowest amplification success was recorded for the *matK* region, while higher rates were observed for the remaining markers, which is consistent with the findings of Karbarz et al. (2024) [37]. The low amplification efficiency of the *matK* region may result from the high variability of DNA sequences where primers bind during the PCR process. The *matK* sequence is approximately 900 bp long, which makes it more susceptible to degradation or damage. This issue occurs not only in Orchidaceae but also in other flowering plants [4,43].

Sequences longer than 200 nucleotides were analyzed using Bioedit 7.2 software and then compared with the BOLD Systems reference database. Based on the database analysis, Table 2 was prepared, showing the number of available reference sequences and barcodes for each species [44]. Three markers (*matK*, *rbcL*, ITS) were analyzed using the new version of the BOLD Systems database, v.5. The database has proven to be very fast and intuitive to use. It allows for the simultaneous transfer of up to 1000 sequences, which significantly improves data analysis and is particularly useful in CITES control activities. The system offers advanced identification features, analysis modes and access to extensive data. Version v.5 is still under development, and some features are not yet active [42].

The results are presented in Table 3, Table 4 and Table 5 for each barcode, which facilitates comparison of their effectiveness in identification. The ITS marker showed the highest effectiveness in assigning to the genus (91%), followed by *matK* (90%). In contrast, the *rbcL* marker did not assign any species to the genus, only to the subfamily Epidendroideae. The ITS barcode was the only one to identify sequences to the species (*P. pelecaniceps*). In the case of the *matK* barcode, the confidence ranged from 81.02 to 91.76 (Table 3) for *Masdevaliia*. For the ITS marker, this range was 93.81 to 100% (Table 5).

The highest identification efficiency at the genus level was achieved for the ITS region (91%). Despite the poor representation of *Masdevallia* species in the BOLD Systems v.5 database [42], the ITS marker effectively assigned most of the analyzed sequences to the correct genus, which proves its high discriminatory power, even with limited reference data. For the ITS marker, the confidence values for assignments to *Masdevallia* ranged from 93.81% to 100%, indicating very high similarity of the sequences to the reference data. The number of supporting records for these assignments was also high (from 5 to 23), which means that the assignments were based on a solid number of comparisons with previously deposited sequences. For comparison, the *matK* marker showed lower confidence values (81.02–91.76%) and a similar range of supporting records (20–22), indicating moderate identification effectiveness. In contrast, despite 100% amplification success, *rbcL* did not assign any sequences to *Masdevallia*, but only to the subfamily *Epidendroideae*, confirming its limited diagnostic value in the analyzed case.

The use of the ITS marker enabled the identification of *Phloeophila pelecaniceps* at the species level, whereas *matK* was unable to resolve taxa below the subfamily level. As no similar studies on *Masdevallia*/*Phloeophila* were available, the results were compared with studies on other orchid genera. Chattopadhyay et al. (2017) reported that the 18S-ITS1-5.8S-ITS2-28S nrDNA locus showed the highest species discrimination ability (95.23%), while *matK* exhibited lower resolution (52%) and acted above the genus level [45]. Similarly, Raskoti and Ale (2021) found that ITS achieved the best identification performance for medicinal orchids (91.62% in Best Match and 90.83% in Best Close Match analyses), confirming its superior species-level resolution compared to *matK* (71.72% and 71.65%, respectively). The low resolution of the plastid genome may result from a lower substitution rate compared to the nuclear genome [18].

This study not only assessed the effectiveness of DNA markers, but also enriched the BOLD Systems database with new sequences for all analyzed *Masdevallia* species, including species not previously present in the database: *M. picea*, *M. stenorhynchos*, *M. cuprea* and *M. schlimii* [44]. This is particularly important for increasing the identification capabilities of the system and supporting the conservation of endangered species.

According to current CITES documents, specimens should be identified at the species, genus and, if necessary, even family level [27]. The developed protocol meets these requirements and can be implemented by institutions involved in the control of trade in protected species.

One of the weaknesses of the study was the limited number of *Masdevallia* species available in the orchid greenhouse of Łańcut Museum-Castle, which restricted the scope of the analyses. The next stages of validating the proposed protocol in a real-world environment should involve extending the analyses to other orchid *Masdevallia* species, with particular emphasis on the ITS marker.

## 4. Conclusions

This study presents the first rapid and practical DNA barcoding protocol for identifying *Masdevallia* orchids in support of CITES enforcement. The ITS marker is recommended as the primary tool for genus-level identification, with *matK* as a complementary option. The protocol enriches reference databases and lays the foundation for broader validation across the genus in real-world conditions.

## Figures and Tables

**Table 1:** Thermocycler parameters in PCR used for different pairs of markers.

Region	Cycle Stage	Initial Denaturation	Denaturation	Annealing	Elongation	Final Elongation
*matK*	Temperature [°C] Time Number of cycles	94 4 min 1×	94 1 min 32×	48 40 s 32×	72 50 s 32×	72 7 min 1×
*rbcL*	Temperature [°C] Time Number of cycles	94 1 min 1×	94 30 s 35×	52 1 min 35×	72 1 min 35×	72 7 min 1×
ITS	Temperature [°C] Time Number of cycles	95 4 min 1×	94 45 s 35×	59 1 min 35×	72 1 min 35×	72 7 min 1×

**Table 2:** Number of sequences and barcodes deposited in the BOLD Systems database [44].

Species	Sequences BOLD Systems	Barcodes > 500 bp
*M. pelecaniceps* (*P. pelecaniceps*) *	3	1
*M. floribunda*	5	4
*M. picea*	0	0
*M. schroederiana*	1	0
*M.* *coccinea*	4	1
*M. stenorhynchos*	0	0
*M. herradurae*	1	0
*M. nidifica*	4	2
*M. civilis*	1	0
*M. cuprea*	0	0
*M. schlimii*	0	0

* Currently classified as *P. pelecaniceps*, it previously belonged to the genus *Masdevallia*.

**Table 3:** DNA barcoding results using BOLD Systems. V. 5 for the *matK* barcode [42].

Sequenced Orchids	*matK*					
	Sequence ID *	Process ID *	Tax Rank	TaxName	Confidence *	Supporting Recs *
*M. pelecaniceps*	M.pelecaniceps_matK	MASD002-25	SUBFAMILY	Epidendroideae	100	10
*M. floribunda* var. *Galleottiana*	M.floribunda_matK	MASD003-25	GENUS	*Masdevallia*	81.24	20
*M. picea*	M.picea_matK	MASD004-25	GENUS	*Masdevallia*	87.82	21
*M. schroederiana*	M.schroederiana_matK	MASD005-25	GENUS	*Masdevallia*	82.72	20
*M. coccinea* var. *harryana*	M.coccineaharryana_matK	MASD006-25	GENUS	*Masdevallia*	81.64	20
*M. coccinea* f. *alba* Hermans	M.coccineaalba_matK	MASD007-25	GENUS	*Masdevallia*	81.02	20
*M. herradurae*	M.herradurae_matK	MASD008-25	GENUS	*Masdevallia*	84.61	21
*M. nidifica*	M.nidifica_matK	MASD009-25	GENUS	*Masdevallia*	91.76	22
*M. civilis*	M.civilis_matK	MASD010-25	GENUS	*Masdevallia*	84.82	21

* Sequence ID—sequence identifier assigned by the database user, Process ID—a unique code assigned to a newly deposited DNA sequence, Confidence—similarity of the sequence assigned to the taxon (%), Supporting Recs—supporting records that provide additional evidence for taxonomic identification.

**Table 4:** Results of DNA barcoding using BOLD Systems. V. 5 for the *rbcL* barcode [42].

Sequenced Orchids	*rbcL*					
	Sequence ID *	Process ID *	Tax Rank	TaxName	Confidence *	Supporting Recs *
*M. pelecaniceps*	M.pelecaniceps_rbcL	MASD012-25	SUBFAMILY	Epidendroideae	100	9
*M. floribunda* var. *Galleottiana*	M.floribunda_rbcL	MASD013-25	SUBFAMILY	Epidendroideae	100	8
*M. picea*	M.picea_rbcL	MASD014-25	SUBFAMILY	Epidendroideae	100	9
*M. schroederiana*	M.schroederiana_rbcL	MASD015-25	SUBFAMILY	Epidendroideae	100	9
*M. coccinea* var. *harryana*	M.coccineaharryana_rbcL	MASD016-25	SUBFAMILY	Epidendroideae	100	8
*M. coccinea* f. *alba Hermans*	M.coccineaalba_rbcL	MASD017-25	SUBFAMILY	Epidendroideae	100	8
*M. stenorhynchos*	M.stenorhynchos_rbcL	MASD018-25	SUBFAMILY	Epidendroideae	100	10
*M. herradurae*	M.herradurae_rbcL	MASD019-25	SUBFAMILY	Epidendroideae	100	10
*M. nidifica*	M.nidifica_rbcL	MASD020-25	SUBFAMILY	Epidendroideae	100	8
*M. civilis*	M.civilis_rbcL	MASD021-25	SUBFAMILY	Epidendroideae	100	8
*M. cuprea*	M.cuprea_rbcL	MASD022-25	SUBFAMILY	Epidendroideae	100	8
*M. schlimii*	M.schlimii_rbcL	MASD023-25	SUBFAMILY	Epidendroideae	100	8

* Sequence ID—sequence identifier assigned by the database user, Process ID—a unique code assigned to a newly deposited DNA sequence, Confidence—similarity of the sequence assigned to the taxon (%), Supporting Recs—supporting records that provide additional evidence for taxonomic identification.

**Table 5:** Results of DNA barcoding using BOLD Systems. V. 5 for ITS barcode [42].

Sequenced Orchids		ITS				
	Sequence ID *	Process ID *	Tax Rank	TaxName	Confidence *	Supporting Recs *
*M. pelecaniceps*	M.pelecaniceps_ITS	MASD001-25	SPECIES	*Phloeophila pelecaniceps*	100	2
*M. picea*	M.picea_ITS	MASD024-25	GENUS	*Masdevallia*	100	5
*M. schroederiana*	M.schroederiana_ITS	MASD025-25	GENUS	*Masdevallia*	97.44	9
*M. coccinea* var. *harryana*	M.coccineaharryana_ITS	MASD026-25	GENUS	*Masdevallia*	100	18
*M. coccinea* f. *alba* Hermans	M.coccineaalba_ITS	MASD027-25	GENUS	*Masdevallia*	100	15
*M. stenorhynchos*	M.stenorhynchos_ITS	MASD028-25	GENUS	*Masdevallia*	95.47	23
*M. herradurae*	M.herradurae_ITS	MASD029-25	GENUS	*Masdevallia*	100	8
*M. nidifica*	M.nidifica_ITS	MASD030-25	GENUS	*Masdevallia*	97.8	12
*M. civilis*	M.civilis_ITS	MASD031-25	GENUS	*Masdevallia*	100	15
*M. cuprea*	M.cuprea_ITS	MASD032-25	GENUS	*Masdevallia*	94.8	12
*M. schlimii*	M.schlimii_ITS	MASD033-25	GENUS	*Masdevallia*	93.81	13

* Sequence ID—sequence identifier assigned by the database user, Process ID—a unique code assigned to a newly deposited DNA sequence, Confidence—similarity of the sequence assigned to the taxon (%), Supporting Recs—supporting records that provide additional evidence for taxonomic identification.

## Data Availability

Data are deposited to BOLD Systems database. Project Code: MASD.
